# Intramolecular Folding of PolyT Oligonucleotides Induced by Cooperative Binding of Silver(I) Ions

**DOI:** 10.3390/molecules27227842

**Published:** 2022-11-14

**Authors:** Jinghua Hao, Dong Cao, Qiang Zhao, Dapeng Zhang, Hailin Wang

**Affiliations:** 1 School of Environment, Hangzhou Institute for Advanced Study, UCAS, Hangzhou 310024, China; 2 State Key Laboratory of Environmental Chemistry and Ecotoxicology, Research Center for Eco-Environmental Sciences, Chinese Academy of Sciences, Beijing 100085, China; 3 University of Chinese Academy of Sciences, Beijing 100049, China

**Keywords:** cooperative binding, DNA, intramolecular folding, metal-mediated base pairs, silver ions

## Abstract

Ag^+^-bridged T-Ag^+^-T was recently discovered in a Ag^+^-DNA nanowire crystal, but it was reported that Ag^+^ had little to no affinity to T nucleobases and T-rich oligonucleotides in solution. Therefore, the binding mode for the formation of this type of novel metallo base pair in solution is elusive. Herein, we demonstrate that Ag^+^ can interact with polyT oligonucleotides once the concentration of Ag^+^ in solution exceeds a threshold value. The threshold value is independent of the concentration of the polyT oligonucleotide but is inversely proportional to the length of the polyT oligonucleotide. The polyT oligonucleotides are intramolecularly folded due to their positively cooperative formation and the stack of T-Ag^+^-T base pairs, resulting in the 5′- and 3′-ends being in close proximity to each other. The intramolecular Ag^+^-folded polyT oligonucleotide has a higher thermal stability than the duplex and can be reversibly modulated by cysteine.

## 1. Introduction

Hydrogen bonds between nucleobases can produce diverse DNA structural motifs depending on the DNA sequences and certain conditions, including B-DNA, Z-DNA, A-DNA, H-DNA, hairpin, triplex, G-quadruplex, and i-motif [1,2]. In most cases, the formation of a particular DNA structure is strongly related with the presence of a specific metal ion. The interactions between nucleic acids and metal ions have been intensively studied by biochemical scientists [3,4,5,6]. At physiological pH, positively charged alkali, alkali earth, and some transition metal ions can interact extensively with the negatively charged phosphate backbone of nucleic acid through electrostatic attraction. However, it is reported that Ag^+^, Hg^2+^, and Pt^2+^ can specifically interact with natural nucleobases through coordination to form metallo base pairs [7].

In metallo base pairs, the hydrogen bonds between complementary nucleobases are formally replaced by coordination bonds to a metal ion between the nucleobases inside the duplex. It has been proven that some metal ions can mediate natural nucleobases (including uracil) to form homo- or hetero-metallo base pairs [3,8,9,10,11,12,13,14,15,16]. From a historical point of view, the T-Hg^2+^-T base pair is considered as the first metal-mediated natural base pair [17]. In order to bring diversity into the field of different metal-modified nucleic acids, a great number of artificial nucleobases have been developed to be incorporated into DNA over the last two decades, such as imidazole, triazole, hydroxypyridone, and salen [3,8,9,10,11,12,13,14,15,16,18,19,20,21]. Recently, the focus has changed from discovering new artificial nucleobases and different metal ions in the DNA duplex to developing the analytical applications for such metal-modified nucleic acids. A variety of applications have been proposed for nucleic acids bearing metal-mediated base pairs, such as sensors for metal ions [9,11,22], redox-sensitive control of DNA structure [23], modulation of polymerase activity [24,25], generation of noble metal nanoclusters [26,27], improvement in the charge transfer capability of DNA duplexes [28], design of metal-responsive DNA machines [29], logic gates [30], DNA magnets [31], and metallo-DNA nanowires [32,33]. Furthermore, extensive studies of metal-mediated base pairs formed by natural and artificial bases potentially expand the genetic alphabet and the design of novel DNA structures and functional DNA molecules [8].

Silver compounds have been used as antibacterial and antifungal agents since ancient times [34]. Though the mechanism of its bioactivity has not been fully understood, one possible mechanism is speculated to be the interaction of Ag^+^ with nucleic acids. Thus, Ag^+^-mediated natural base pairs have been one of the intensive research fields among metal-mediated base pairs, especially the canonical C-Ag^+^-C base pair [11,35,36]. Since C-Ag^+^-C was first reported by the Ono group in 2008 [35], several other Ag^+^-mediated natural base pairs have been recently identified and studied through theoretical calculation and different experimental techniques, namely, C-Ag^+^-T, C-Ag^+^-A, G-Ag^+^-G, T-Ag^+^-T, and A-Ag^+^-A [27,32,37,38,39,40,41,42,43,44,45]. With regards to T-Ag^+^-T, the structure of this novel Ag^+^-mediated natural homo base pair was well characterized by X-ray crystallography of the crystal of a silver-DNA hybrid nanowire [32]. However, it is reported that Ag^+^ has a very low affinity to the T nucleobase and T-rich/polyT oligonucleotides in solution [41,46,47,48]. Therefore, it is unclear whether Ag^+^ can bridge the T nucleobases of DNA to form T-Ag^+^-T base pairs in solution, and what the binding mode is.

Herein, a 16-mer polyT (pT16) oligonucleotide, which could remove the constraints of Watson–Crick base pairs in DNA, was firstly designed to investigate the formation mechanism of Ag^+^-mediated T-Ag^+^-T base pairs in a neutral pH solution. We studied the effect of Ag^+^ on the pT16 oligonucleotide conformation by UV absorption spectroscopy, circular dichroism (CD) spectroscopy, and fluorescence spectroscopy. Moreover, another three polyT oligonucleotides (pT4, pT8, pT32) were used to investigate the effect of chain length on their interaction with Ag^+^ to form T-Ag^+^-T. In addition, mass spectrometry (MS) and fluorescence spectroscopy based on molecular beacons and light-switching probes were employed to understand the nature of the conformational change in polyT oligonucleotides induced by the formation of T-Ag^+^-T base pairs. Thermal denaturation was used to test the effect of the incorporation of Ag^+^ ions on the thermal stability of polyT oligonucleotides. Finally, the reversibility of Ag^+^-induced folding of polyT oligonucleotides was studied using the well-known Ag^+^ chelator L-cysteine.

## 2. Results and Discussions

### 2.1. Folding of PolyT Oligonucleotides Induced by Ag^+^ via Cooperative Formation of T-Ag^+^-T

**Figure 1:**
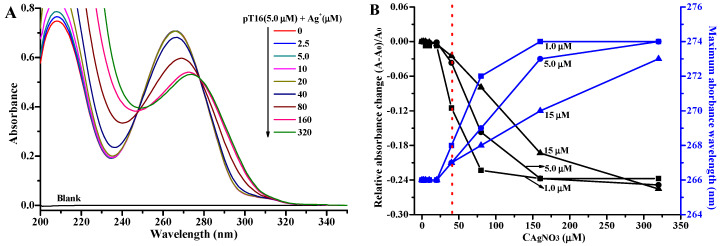
(**A**) UV absorption spectra of 5.0 μM pT16 in the absence and presence of 2.5, 5.0, 10, 20, 40, 80, 160, and 320 μM AgNO_3_. (**B**) Plots of the relative absorbance change (left, black) and wavelength (right, blue) at the maximum absorption against the concentration of AgNO_3_ for pT16 solutions at 1.0 μM (square), 5.0 μM (circle), and 15 μM (uptriangle). The samples were prepared in 10 mM Tris-HAc buffer pH 7.0. *A*_0_ and *A* are the absorbance at maximum absorption of the oligonucleotide solutions in the absence and presence of AgNO_3_, respectively.

The UV absorbance of DNA is usually dependent on its conformation; thus, UV spectroscopy can be used to elucidate whether the structure of DNA is folded upon its binding with ligands [23,49]. In order to investigate whether Ag^+^ can interact with DNA and induce its conformation change through the formation of novel Ag^+^-mediated homo base pairs T-Ag^+^-T, the effect of AgNO_3_ on the absorbance of a pT16 oligonucleotide (Table 1) at pH 7.0 was studied. The pT16 oligonucleotide exists in a random coiled structure in an aqueous solution at neutral pH. Moreover, the constraints of the Watson–Crick hydrogen bond can be removed upon formation of Ag^+^-bridged T-Ag^+^-T base pairs. The UV absorption spectra of 5.0 μM pT16 solutions with increasing concentrations of AgNO_3_ display an obvious hypochromic effect, accompanied by a bathochromic shift (from 266 to 274 nm) in the wavelength at maximum absorbance (λ_max_; Figure 1). However, it is interesting to find that the absorbances of pT16 solutions hardly change until the concentration of Ag^+^ reaches 40 μM, above which the absorbances dramatically decrease until the concentration of Ag^+^ reaches 160 μM, where the absorbance begins to level off (Figure 1). The relative absorbance change of pT16 at λ_max_ in the presence of 160 μM AgNO_3_ (−23.7%) is close to the relative absorbance change at 260 nm for hybridizing pT16 with equimolar 16-mer polyA (pA16, Table 1) to form duplex DNA (−25.4%; Figure S1) [50]. In addition, we investigated the effect of HgCl_2_ on the absorbance of 5.0 μM pT16 upon formation of canonical T-Hg^2+^-T base pairs. As shown in Figure S2A,B, the absorbance of pT16 solutions decrease linearly as the concentration of Hg^2+^ increases to 40 μM (−25.5%, T nucleobase: Hg^2+^ = 2:1), accompanied by a bathochromic shift, and then become flat with the further increasing concentration of Hg^2+^. The results suggest that Ag^+^ can interact with polyT oligonucleotides in the presence of a high concentration of AgNO_3_ in solution and result in T nucleobases that stack well, which may be through the formation of T-Ag^+^-T base pairs similar to the formation of T-Hg^2+^-T between polyT and Hg^2+^ [51].

We further studied the effect of AgNO_3_ on the absorbance of pT16 at solution concentrations of 1.0 μM (Figure S3A) and 15 μM (Figure S3B). Although the difference in concentration is more than one order of magnitude, interestingly, the absorbances of both pT16 solutions remain unchanged until the concentration of Ag^+^ in solution reaches 40 μM, and then there is an obvious decrease and levelling off with further increasing the concentration of Ag^+^ (Figure 1B). This phenomenon is the same as above 5.0 μM pT16 titrated with AgNO_3_. The results indicate that the interaction between Ag^+^ and pT16 can happen when the concentration of Ag^+^ achieves a threshold value of 40 μM, which is independent of the concentration of pT16. Furthermore, the sigmoidal Ag^+^ binding curves of 1.0 μM and 5.0 μM pT16 fit well with the Hill–Langmuir equation (R^2^ = 0.999, Figure S4), which is useful for determining the degree of cooperativity of the ligand binding to the macromolecules. The obtained Hill coefficients (*n*) are 4.33 and 3.39 for 1.0 and 5.0 μM pT16, respectively, indicating the positively cooperative binding (*n* > 1) of Ag^+^ to pT16 [52]. Therefore, the results imply that silver(I) ions are incorporated into pT16 to form T-Ag^+^-T in a positive cooperativity fashion.

**Figure 2:**
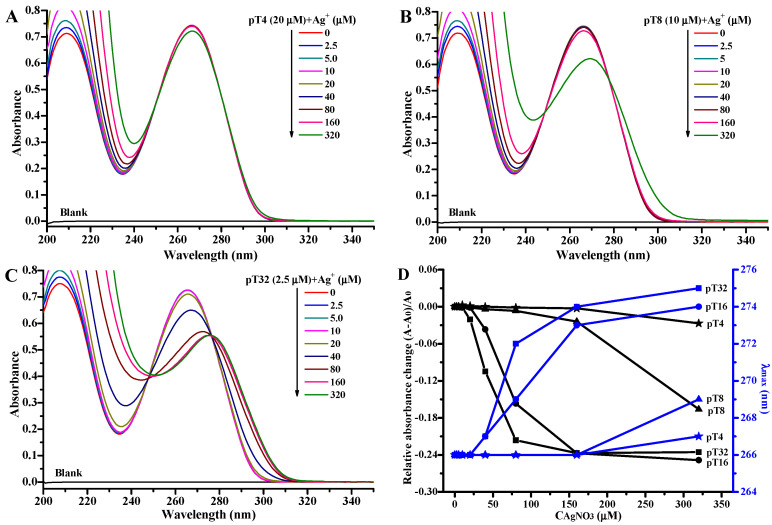
UV absorption spectra of 20 μM T4 (**A**), 10 μM T8 (**B**), and 2.5 μM T32 (**C**) solutions in the absence and presence of 2.5, 5.0, 10, 20, 40, 80, 160, and 320 μM AgNO_3_. (**D**) Plots of absorbance change (left, black) and wavelength (right, blue) at the maximum absorption against the concentration of AgNO_3_ for 20 μM T4 (star), 10 μM T8 (uptriangle), 5.0 μM T16 (circle), and 2.5 μM T32 (square). The conditions are the same as those for Figure 1.

In order to know whether the cooperative formation of T-Ag^+^-T is related to the length of the polyT (pT_n_) oligonucleotides, we additionally designed two shorter oligonucleotides, pT4 and pT8, and a longer oligonucleotide, pT32 (Table 1), and investigated the change in absorbance for these three oligonucleotides titrated with AgNO_3_ via two approaches. One method was to fix the strand concentrations of these oligonucleotides at 5.0 μM (Figure S5A–C), and the other was to fix the concentrations of T nucleobase of these oligonucleotides at 80 μM (i.e., 20 μM pT4, 10 μM pT8, and 2.5 μM pT32; Figure 2A–C). No matter which approach was used, the changes in the absorbance and wavelength at maximum absorption are similar for these three pTn oligonucleotides with different length (Figure 2D and Figure S5D). The Ag^+^ concentration at which the absorbance starts to decrease is inversely proportional to the length of the oligonucleotides: 320, 160, 40, and 20 μM for pT4, pT8, pT16, and pT32, respectively. The Hill coefficient (*n*) is 3.48 for 2.5 μM pT32 (Figure S6). The results suggest that the interaction threshold value of Ag^+^ decreases with the increase in the length of polyT oligonucleotides, and further confirm that the interaction threshold value of Ag^+^ is independent of the concentration of polyT oligonucleotides.

**Figure 3:**
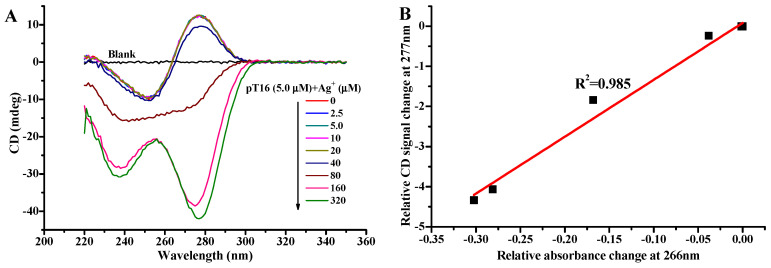
(**A**) CD spectra of 5.0 μM pT16 in the absence and presence of 2.5, 5.0, 10, 20, 40, 80, 160, and 320 μM AgNO_3_. (**B**) The relationship between the relative CD signal change at 277 nm and the absorbance change at 266 nm. The reaction conditions are the same as those for Figure 1.

CD spectroscopy is a valuable tool for providing important information about the conformational properties of DNA molecules [53]. We employed CD spectrometry to study the effect of AgNO_3_ on folding the conformation of pT16 oligonucleotide. The CD spectrum of pT16 has a positive peak at 277 nm and a negative peak at 251 nm in the absence of Ag^+^ (Figure 3A), which is consistent with the published literature on T-rich oligonucleotides of a primarily unfolded conformation [33,51,53]. The CD spectra of pT16 solutions begin to change until the concentration of Ag^+^ achieves 40 μM, and then remarkably change with further increases in the concentration of Ag^+^ (Figure 3A), eventually, presenting a new negative peak at around 277 nm. Moreover, the relative change in the CD signal at 277 nm due to the increase in the concentration of Ag^+^ is proportional to the relative absorbance change in pT16 at 266 nm (Figure 3B). These results are consistent with those from UV absorbance and confirm that the polyT oligonucleotides are folded by the positively cooperative formation of T-Ag^+^-T base pairs.

### 2.2. Intramolecular Folding of PolyT Oligonucleotides Induced by Ag^+^

**Figure 4:**
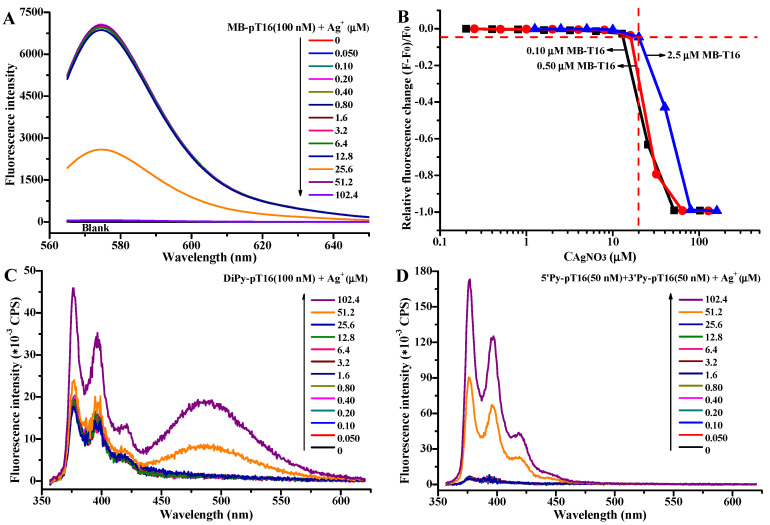
Fluorescence emission spectra of 100 nM MB-pT16 (**A**), 100 nM DiPy-pT16 (**C**), and the 50 nM equimolar mixture of 5′Py-pT16 with 3′Py-pT16 (**D**) in the absence and presence of 0.050, 0.10, 0.20, 0.40, 0.80, 1.6, 3.2, 6.4, 12.8, 25.6, 51.2, and 102.4 μM AgNO_3_. (**B**) Plots of the relative fluorescence change in TMR at 578 nm against the concentration of AgNO_3_ for 100 nM (black square), 500 nm (red circle), 2500 nM (blue uptriangle) MB-pT16. The reaction conditions are the same as those for Figure 1. The excitation wavelengths for TMR and pyrene were set at 557 and 347 nm, respectively. The excitation and emission slit bandwidths were both set at 5 nm. *F*_0_ and *F* were the fluorescence intensities of MB-pT16 at 578 nm in the absence and presence of AgNO_3_, respectively.

Molecular beacons (MB) have been used for the development of DNA sensors and for monitoring DNA–ligand interactions [54]. To fully understand the conformational change in polyT oligonucleotides induced by the Ag^+^-mediated formation of T-Ag^+^-T base pairs, we designed a molecular beacon of pT16 (MB-pT16; Table 1), labeled with a tetramethylrhodamine (TMR) fluorophore and a Black Hole Quencher 2 (BHQ2) at the 5′- and 3′-ends of pT16, respectively. The fluorescence intensity of TMR does not change until the concentration of Ag^+^ is higher than 20 μM for all three MB-pT16 solution concentrations of 100 nM (Figure 4A), 500 nM (Figure S7A), and 2.5 μM MB-pT16 (Figure S7B). The fluorescence intensity of TMR dramatically decreases at higher Ag^+^ concentrations and approaches steady states with the further addition of AgNO_3_ (Figure 4A,B and Figure S7). The threshold value of Ag^+^ obtained by fluorescence (~20 μM) is slightly lower than that obtained by UV spectroscopy (40 μM), which may be due to the fact that fluorescence is a more sensitive technique. Otherwise, the results are not only consistent with the UV absorption and CD results, but also suggest that the 5′- and 3′-termini of polyT oligonucleotides are close to each other after the cooperative formation of T-Ag^+^-T.

To determine whether both ends of the MB-pT16 probe are in close proximity via the formation of either intramolecular or intermolecular T-Ag^+^-T modes, another two probes were designed, in which TMR and BHQ2 were individually labeled at the 5′-(5′TMR-pT16) and 3′-(3′BHQ2-pT16) termini of pT16, respectively (Table 1). When an equimolar mixture of the two probes was titrated with AgNO_3_ solution, the fluorescence intensity of 5′TMR-pT16 would be quenched to about half by 3′BHQ2-pT16 if the T-Ag^+^-T base pairs were formed via intermolecularly folding pT16. Conversely, the fluorescence intensity of 5′TMR-pT16 would not change in the event of intramolecular formation of the T-Ag^+^-T base pairs, whether mixed with quencher labeled or unlabeled pT16. The fluorescence change in the 50 nM 5′TMR-pT16 solution titrated with AgNO_3_ is almost the same when it is mixed with equimolar unlabeled pT16 or with equimolar 3′BHQ2-pT16 (Figure S8A,B), though the relative fluorescence of TMR at 578 nm slightly decreases in the presence of a high concentration of AgNO_3_ (~27% at 102.4 μM AgNO_3_; Figure S8C). Collectively, the results show that polyT oligonucleotides are intramolecularly folded via cooperative formation of T-Ag^+^-T.

It is known that pyrene is a spatially sensitive fluorescent dye, which can form excimer (excited-state dimer) upon close encounters and emits fluorescence at longer wavelengths than monomer [55]. This characteristic of the distance-dependent excimer formation of pyrene has been exploited for the DNA single nucleotide polymorphisms detection, DNA-binding targets sensing and DNA structure analysis [56,57,58]. To further confirm the cooperative formation of intramolecular T-Ag^+^-T in polyT oligonucleotide, we designed and synthesized another three pT16 oligonucleotide probes having pyrene moieties at their 5′- and/or 3′-ends (DiPy-pT16, 5′Py-pT16, and 3′Py-pT16; Table 1). The effects of AgNO_3_ on the fluorescence of 100 nM DiPy-pT16 and the 50 nM equimolar mixture of 5′Py-pT16 and 3′Py-pT16 were investigated in the same manner as that for the above turn-off molecular beacon. As shown in Figure 4C,D, the monomer emission peaks (at 376 and 397 nm) are observed, which exhibit almost no change when the concentration of Ag^+^ is lower than the threshold value (40 μM) obtained by UV experiments. However, a new excimer peak appears at 485 nm for the DiPy-pT16 probe when the concentration of Ag^+^ is higher than 40 μM in solution, accompanying a slight increase in monomer fluorescence intensity. With regards to the equimolar mixture of 5′Py-pT16 and 3′Py-pT16, no excimer peak is observed, even though the monomer fluorescence intensities increase sharply in the presence of 51.2 and 102.4 μM AgNO_3_. The results for this distance-dependent light-switching probe further confirm that Ag^+^ ions are cooperatively incorporated into pT16 via an intramolecular interaction pattern.

**Figure 5:**
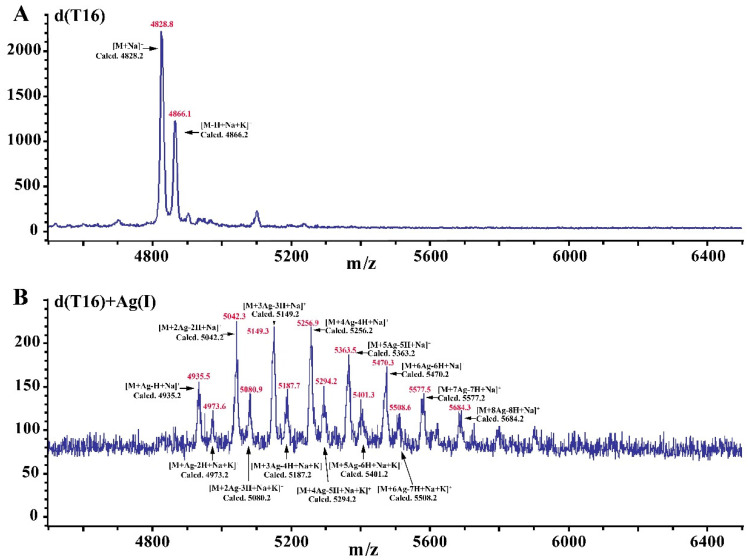
Positive MALDI-TOF/MS spectra of 100 μM pT16 in the absence (**A**) and presence (**B**) of 800 μM AgNO_3_. The reaction conditions are the same as those for Figure 1.

MS is a powerful tool for probing noncovalent interaction between DNA and ligands, which can provide the ligand binding stoichiometry [59,60]. To further test and verify that intramolecular T-Ag^+^-T may be formed, matrix-assisted laser desorption/ionization time-of-flight mass spectrometry (MALDI-TOF MS) was used to characterize the complex formed between polyT oligonucleotides and Ag^+^ in the positive ion mode. As shown in Figure 5A, sodium-adduct [M+Na]^+^ and deprotonated sodium–potassium-adduct [M−H+Na+K]^+^ molecular ions are formed for 100 μM pT16 in the absence of AgNO_3_. This is a common phenomenon because nucleic acids are prone to forming sodium and potassium adducts in MALDI MS [61]. When 100 μM pT16 was mixed with 800 μM AgNO_3_ (Ag^+^/base = 0.5), peaks to the complexes of one pT16-metal adduct with different numbers of Ag^+^ ions ([M+*n*Ag−*n*H+Na]^+^ and [M+*n*Ag−(*n*+1)H+Na +K]^+^) are observed with *n* ranging from 1 to 8 (Figure 5B). There are no signals at the mass/charge ratio (*m*/*z*) nearby to 9000 (Figure S9A), which would correspond to the complexes consisting of two pT16 molecules and a different number of Ag^+^ ions. For pT8, we similarly observe the peaks to the complexes of one pT8 molecule and 1−4 Ag^+^ ions (Figure S9B,C). The results prove that Ag^+^ ions interact with polyT oligonucleotides to probably form T-Ag^+^-T base pairs via an intramolecular binding fashion.

**Figure 6:**
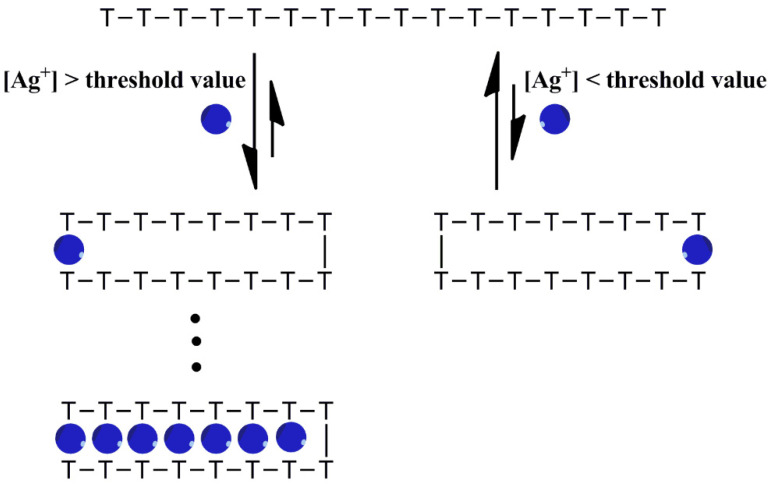
Schematic representation of positively cooperative formation of Ag^+^-bridged intramolecular T-Ag^+^-T base pairs in polyT oligonucleotides.

Based on our above experimental data and discussion, a binding mode of formation T-Ag^+^-T base pairs in polyT oligonucleotides induced by Ag^+^ in solution is conveyed as the following (Figure 6): Ag^+^ ions are incorporated into polyT oligonucleotides with significant difficulty when the concentration of Ag^+^ is lower than the threshold value. However, intramolecular T-Ag^+^-T base pairs would be dramatically formed in a positively cooperative binding manner once the concentration of Ag^+^ in solution is higher than the threshold value, which is independent of the concentration of polyT oligonucleotides. Moreover, the threshold value is inversely proportional to the length of the polyT oligonucleotides, which may be due to the flexibility of the polyT oligonucleotides. It is known that the single strand DNA becomes more flexible as the length of DNA increases [62,63], which probably make it easier to intramolecularly fold polyT. In addition, the threshold value of Ag^+^ is 20–320 μM for polyT oligonucleotides with a length ranging from 4 to 32 bases, so it would be difficult to observe Ag^+^ binding with T nucleobase and T-rich/polyT oligonucleotides in solution when the investigated concentration of Ag^+^ is lower than the threshold value.

### 2.3. The Stability of the Intramolecular Ag^+^-Folded PolyT Oligonucleotide

**Figure 7:**
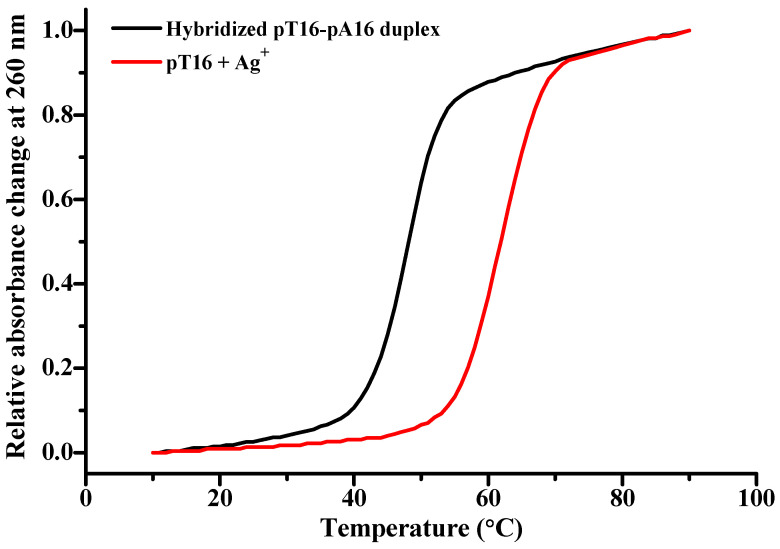
Thermal denaturation curves of 2.5 μM hybridized pT16-pA16 duplex (black) and 5.0 μM pT16 in the presence of 320 μM AgNO_3_ (red). The sample for pT16 under Ag^+^ was prepared in 10 mM Tris-HAc buffer at pH 7.0, and the hybridized pT16-pA16 duplex in 10 mM Tris-HCl, 50 mM NaCl, 10 mM MgCl_2_ pH 7.5.

Thermal denaturation methods are widely used to characterize the stability of the duplex DNA containing natural base pairs or/and metal-mediated base pairs [14,64]. To determine the thermal stability of the intramolecular polyT-Ag^+^ complex, we measured the melting temperature (*T_m_*) of pT16 in the presence of Ag^+^. Compared with the *T_m_* of the hybridized duplex between pT16 and complementary pA16 in the presence of 50 mM Na^+^ and 10 mM Mg^2+^ (*T_m_* = 48.1 °C), Figure 7 shows that there is an obvious increase in the melting temperature of pT16 in the presence of 320 μM AgNO_3_ (*T_m_* = 61.9 °C, ∆*T_m_* = 13.8 °C). The results demonstrate that Ag^+^ ions increase the thermal stability of polyT oligonucleotides once cooperative formation of intramolecular T-Ag^+^-T base pairs has occurred.

Though the stability of the intramolecular polyT-Ag^+^ complex is very high, we are interested in knowing whether the Ag^+^-induced folding of polyT is reversible. Cysteine is a well-known Ag^+^ chelator and has been used to inhibit the formation of G-Ag^+^-G base pairs in DNA [65]. MB-pT16 was repeatedly titrated with AgNO_3_ and then L-cysteine solutions. As shown in Figure S10, the fluorescence intensity can be recovered to its initial state upon the addition of L-cysteine to the Ag^+^-folded MB-pT16, indicating that the structure is unfolded. Moreover, multiple cycling is possible by means of investigating the effect of sequential additions of AgNO_3_ and L-cysteine on the fluorescence of MB-pT16. The results suggest that the intramolecular folding of polyT oligonucleotides induced cooperatively by Ag^+^ ions can be reversibly modulated by the addition of L-cysteine.

## 3. Materials and Methods

### 3.1. Chemicals and Reagents

The oligonucleotides used in this work were synthesized by Sangon Biotech Co. Ltd. (Shanghai, China) and purified by ULTRAPAGE or HPLC, some of which were fluorescently labeled with a fluorophore and/or a quencher at the specified position. The qualities of all synthesized oligonucleotides were checked by mass spectrometry. The lyophilized oligonucleotides were initially dissolved in sterilized ultrapure water to about 100 μM as stock solutions and stored in refrigerator at −20 °C. Further dilutions were carried out in the appropriate buffer. The concentration of the oligonucleotides was presented as single-strand form unless otherwise specified, which was determined by the absorbance at 260 nm with Nanodrop 2000 (Thermo Scientific, Wilmington, DE, USA). Tris(hydroxymethyl)aminomethane (Tris) and acetic acid (HAc) were purchased from Amresco (Solon, OH, USA) and Fisher Scientific (Pittsburgh, PA, USA), respectively. AgNO_3_ and HgCl_2_ were obtained from National Pharmaceutical Group Chemical Reagent Co. (Beijing, China). All these chemical reagents were analytical grade and directly used as received without further purification. Ultrapure water with 18.2 MΩ·cm was obtained from a Purelab Ultra Elga Lab water system (VWS Ltd., High Wycombe, Bucks, UK) and was sterilized before using to prepare solution. All work solutions were prepared in Tris-HAc buffer (10 mM, pH 7.0) unless otherwise specified.

### 3.2. UV Spectroscopy Measurement

UV spectroscopy experiments were performed on a SHIMADZU UV-1900 UV-Vis spectrophotometer (Tokyo, Japan) at room temperature. The oligonucleotide samples (2 mL) were diluted to the indicated concentration in 10 mM Tris-HAc buffer at pH 7.0. For Ag^+^ titration experiments, aliquots of AgNO_3_ were added to the aqueous solution and mixed to the desired concentration by using the pipettes. Spectra were recorded over a wavelength range from 350 to 200 nm with scan rate at 200 nm/min and data interval for 1 nm after incubating each sample for 2 min. A 10 mm optical path length quartz cuvette was used for UV-Vis measurement.

### 3.3. CD Spectroscopy Analysis

CD spectra were recorded on a JASCO J-815 spectrophotometer (Tokyo, Japan) at room temperature. DNA solutions (5.0 μM) were prepared in 10 mM Tris-HAc buffer at pH 7.0. Aliquots of AgNO_3_ were added and mixed to the desired concentration in the aqueous solution by using the pipettes, and then the mixtures were incubated at room temperature for 2 min before CD measurements. The recorded wavelengths were set at the range from 220 nm to 350 nm, and the scan rate was set at 100 nm/min. The response time and bandwidth was 1 s and 0.5 nm bandwidth, respectively. Quartz cuvette with 10 mm × 10 mm path length was used for CD measurement. The spectra were averaged over 3 successive scans.

### 3.4. Fluorescence Spectroscopy Assay

The fluorescence measurements were conducted on a JASCO FP-8300 spectrofluorometer (Tokyo, Japan) or a HORIBA Fluoromax-4 spectrofluorometer (Edison, NJ, USA). For TMR dye, the fluorescence spectra were recorded over wavelength range from 565 to 650 nm with an excitation wavelength at 557 nm. For pyrene molecule, the fluorescence spectra were recorded over wavelength range from 357 to 620 nm with an excitation wavelength at 347 nm. The excitation and emission slit bandwidths were both set at 5 nm for 100 nM probe, at 5 nm and 2.5 nm, for 500 nM probe, and both at 2.5 nm for 2.50 μM probe, respectively. The scan rate and response time were set at 200 nm/min and 1.0 s, respectively. The oligonucleotide probe was diluted to the indicated concentration in 10 mM Tris-HAc buffer, pH 7.0. The oligonucleotide probe solution (2 mL) was titrated by directly adding aliquots of Ag^+^ into the quartz cell (10 mm × 10 mm) with micropipette. Fluorescence spectrum was recorded after fully mixing Ag^+^ with the probe solution and incubating for 2 min.

### 3.5. Thermal Melting (T_m_) Analysis

Melting temperatures (*T_m_*) were determined from the changes in absorbance at 260 nm as a function of temperature in a 10 mm path length quartz cuvette on a SHIMADZU UV-1900 UV-Vis spectrophotometer equipped with a temperature control system. Solutions of Ag^+^-folded polyT16 in 10 mM Tris-HAc, pH 7.0 aqueous buffer was firstly equilibrated at 10 °C for 5 min, and then slowly ramped to 90 °C with 1 °C steps at rate of 1 °C/min. *T_m_* values were calculated as the first derivatives of the heating curves. 

### 3.6. Mass Spectroscopy (MS) Detection

Matrix-assisted laser desorption/ionization mass spectrometry (MALDI-TOF-MS) experiments were performed on a Bruker Autoflex III Smartbeam mass spectrometer (Bruker Daltonics, Bremen, Germany). The matrix was prepared by dissolving 15 mg anthranilic acid (AA), 7.5 mg nicotinic acid (NA), and 30 µL 10 mM diammonium hydrogen citrate (DHC) in 250 µL acetonitrile and 150 µL sterilized ultrapure water. The samples were pretreated by mixing the pT16 or pT8 (100 μM) without/with AgNO_3_ (800 or 400 μM) in 10 mM Tris-HAc buffer at pH 7.0. Then the sample was firstly deposited on a 384 Anchor-Chip MALDI target plate (Bruker Daltonics, Billerica, MA, USA). After the samples were dried, the same volume of matrix was deposited on the sample for crystallization. The recorded mass to charge (m/z) was set at the range from 3000 to 10,000 in the linear positive ion mode, and the laser energy was set at 76%. For each measurement, mass spectrometry data were obtained by 2000 laser shots.

### 3.7. Hill–Langmuir Equation

The Hill–Langmuir equation is useful in determining the degree of cooperativity of the ligand binding to the macromolecule. The obtained Hill coefficient (n) provides a way to quantify the degree of interaction between ligand binding sites. The receptor (R), polyT oligonucleotide here, has *n* potential binding sites for ligand Ag^+^ to bridge formation T-Ag^+^-T base pairs. The binding of Ag^+^ to polyT oligonucleotide can be represented by the chemical equilibrium expression:

R + nL ↔ RLn(1)

The apparent dissociation constant K_d_ is given by:

(2) $$K_{d}=\frac{\left[R\right]\times {\left[L\right]}^{n}}{\left[\mathrm{RLn}\right]}$$

At the same time, θ, the ratio of the concentration of occupied polyT oligonucleotide to total concentration of polyT oligonucleotide, is given by:

(3) $$\theta =\frac{\left[\mathrm{RLn}\right]}{\left[\mathrm{RLn}\right]+\left[R\right]}$$

By using the above expression for the dissociation constant, we can replace [RLn] to yield a simplified expression for θ:

(4) $$\theta =\frac{{\left[L\right]}^{n}}{K_{d}+{\left[L\right]}^{n}}$$

## 4. Conclusions

In summary, we find that Ag^+^ ions can interact with polyT oligonucleotides when the concentration of Ag^+^ in solution is over a threshold value. Ag^+^ ions are incorporated into polyT oligonucleotides via a positively cooperative binding mode to form recently discovered novel T-Ag^+^-T base pairs, which are well stacked similar to Watson–Crick base pairs in duplex DNA and T-Hg^2+^-T in polyT oligonucleotide. Furthermore, fluorescent probe studies and mass spectrometry analysis prove that polyT oligonucleotides are folded by forming intramolecular T-Ag^+^-T base pairs with 5′-and 3′-termini of a single polyT molecule in close proximity. The threshold value for the cooperative formation of T-Ag^+^-T is independent of the concentration of polyT oligonucleotides, but is inversely proportional to the length of polyT oligonucleotides. The thermal stability of Ag^+^-folded polyT oligonucleotides is significantly enhanced once intramolecular T-Ag^+^-T base pairs are cooperatively formed. The structure of the random-coiled and Ag^+^-folded polyT oligonucleotide is reversibly regulated by l-cysteine. This finding is potentially applicable to the study of DNA-based silver nanoclusters and DNA nanotechnology.

## Figures and Tables

**Table 1:** The names, sequences, and modifications of the DNA oligonucleotides presented in this work.

Name	Sequence (from 5′ to 3′)
pT16	TTTTTTTTTTTTTTTT
MB-pT16	TMR-TTTTTTTTTTTTTTTT-BHQ2
5′TMR-pT16	TMR-TTTTTTTTTTTTTTTT
3′BHQ2-pT16	TTTTTTTTTTTTTTTT-BHQ2
DiPy-pT16	Pyrene-TTTTTTTTTTTTTTTT-Pyrene
5′Py-pT16	Pyrene-TTTTTTTTTTTTTTTT
3′Py-pT16	TTTTTTTTTTTTTTTT-Pyrene
pA16	AAAAAAAAAAAAAAAA
pT8	TTTTTTTT
pT4	TTTT
pT32	TTTTTTTTTTTTTTTTTTTTTTTTTTTTTTTT

## Data Availability

Not applicable.

## Supplementary Materials

The following supporting information can be downloaded at: https://www.mdpi.com/article/10.3390/molecules27227842/s1, Figure S1: UV absorption spectra of 2.5 μM pT16 (red curve), 2.5 μM pA16 (green curve) and hybridized mixture (blue curve) of 2.5 μM pT16 with equimolar pA16; Figure S2: (A) UV absorption spectra of 5.0 μM pT16 in the absence and presence of 2.5, 5.0, 10, 20, 40, 80, 160 and 320 μM HgCl_2_. (B) Plots of the relative absorbance change (left, black) and wavelength (right, blue) at the maximum absorption against the concentration of HgCl_2_ for pT16 solutions at 5.0 μM (square); Figure S3: UV absorption spectra of 1.0 μM (A) and 15 μM (B) pT16 in the absence and presence of 2.5, 5.0, 10, 20, 40, 80, 160 and 320 μM AgNO_3_; Figure S4: Plots of the bound fraction against the concentration of AgNO_3_ for 1.0 μM pT16 (black square) and 5.0 μM pT16 (red circle); Figure S5: UV absorption spectra of 5.0 μM pT4 (A), pT8 (B) and pT32 (C) in the absence and presence of 2.5, 5.0, 10, 20, 40, 80, 160 and 320 μM AgNO_3_. (D) Plots of absorbance change (left, black) and wavelength (right, blue) at the maximum absorption against the concentration of AgNO_3_ for 5.0 μM pT4 (star), pT8 (uptriangle), pT16 (circle) and pT32 (square); Figure S6: Plots of the bound fraction against the concentration of AgNO_3_ for 2.5 μM pT32; Figure S7: Fluorescence emission spectra of 500 nM (A) and 2.5 μM (B) MB-pT16 in the absence and presence of 0.5-fold, 1-fold, 2-fold, 4-fold, 8-fold, 16-fold, 32-fold, 64-fold, 128-fold, 256-fold and 512-fold AgNO_3_; Figure S8: Fluorescence emission spectra of the mixture of 50 nM 5’TMR-pT16 with equimolar 3’BHQ2-pT16 (A) or unlabeled pT16 (B) in the absence and presence of 0.050, 0.10, 0.20, 0.40, 0.80, 1.6, 3.2, 6.4, 12.8, 25.6, 51.2 and 102.4 μM AgNO_3_. (C) Plots of the relative fluorescence change of TMR at 578 nm against the concentration of AgNO_3_ for the mixture of 50 nM 5’TMR-pT16 with equimolar 3’BHQ2-pT16 (red circle) or unlabeled pT16 (black square); Figure S9: Positive MALDI-TOF/MS spectra of 100 μM pT16 in the presence of 800 μM AgNO_3_ in a wider molecular weight range (A) and 100 μM pT8 in the absence (B) and presence (C) of 400 μM AgNO.; Figure S10: Cycling of the folding and unfolding of 100 nM MB-pT16 through monitoring the relative fluorescence of TMR at 578 nm.

## References

[1] Choi J., Majima T. (2011). Conformational changes of non-B DNA. Chem. Soc. Rev..

[2] Day H.A., Huguin C., Waller Z.A.E. (2013). Silver cations fold i-motif at neutral pH. Chem. Commun..

[3] Müller J. (2019). Nucleic acid duplexes with metal-mediated base pairs and their structures. Coordin. Chem. Rev..

[4] Zhou W., Saran R., Liu J. (2017). Metal Sensing by DNA. Chem. Rev..

[5] Liu H.-K., Sadler P.J. (2011). Metal Complexes as DNA Intercalators. Acc. Chem. Res..

[6] Boerner L.J.K., Zaleski J.M. (2005). Metal complex–DNA interactions: From transcription inhibition to photoactivated cleavage. Curr. Opin. Chem. Biol..

[7] Berti L., Burley G.A. (2008). Nucleic acid and nucleotide-mediated synthesis of inorganic nanoparticles. Nat. Nanotechnol..

[8] Naskar S., Guha R., Müller J. (2020). Metal-Modified Nucleic Acids: Metal-Mediated Base Pairs, Triples, and Tetrads. Angew. Chem. Int. Ed..

[9] Jash B., Müller J. (2017). Metal-Mediated Base Pairs: From Characterization to Application. Chem. Eur. J..

[10] Lippert B., Miguel P.J.S. (2016). The Renaissance of Metal–Pyrimidine Nucleobase Coordination Chemistry. Acc. Chem. Res..

[11] Tanaka Y., Kondo J., Sychrovský V., Šebera J., Dairaku T., Saneyoshi H., Urata H., Torigoe H., Ono A. (2015). Structures, physicochemical properties, and applications of T-Hg-II-T, C-Ag-I-C, and other metallo-base-pairs. Chem. Commun..

[12] Scharf P., Müller J. (2013). Nucleic Acids with Metal-Mediated Base Pairs and Their Applications. ChemPlusChem.

[13] Takezawa Y., Shionoya M. (2012). Metal-Mediated DNA Base Pairing: Alternatives to Hydrogen-Bonded Watson-Crick Base Pairs. Acc. Chem. Res..

[14] Ono A., Torigoe H., Tanaka Y., Okamoto I. (2011). Binding of metal ions by pyrimidine base pairs in DNA duplexes. Chem. Soc. Rev..

[15] Clever G.H., Shionoya M. (2010). Metal-base pairing in DNA. Coordin. Chem. Rev..

[16] Clever G.H., Kaul C., Carell T. (2007). DNA-metal base pairs. Angew. Chem. Int. Ed..

[17] Katz S. (1962). Reversible Reaction of Double-Stranded Polynucleotides and HgII: Separation of the Strands. Nature.

[18] Tanaka K., Shionoya M. (1999). Synthesis of a novel nucleoside for alternative DNA base pairing through metal complexation. J. Org. Chem..

[19] Takezawa Y., Müller J., Shionoya M. (2017). Artificial DNA Base Pairing Mediated by Diverse Metal Ions. Chem. Lett..

[20] Mandal S., Müller J. (2017). Metal-mediated DNA assembly with ligand-based nucleosides. Curr. Opin. Chem. Biol..

[21] Wagenknecht H.-A. (2003). Metal-mediated DNA base pairing and metal arrays in artificial DNA: Towards new nanodevices. Angew. Chem. Int. Ed..

[22] Ono A., Togashi H. (2004). Highly selective oligonucleotide-based sensor for mercury(II) in aqueous solutions. Angew. Chem. Int. Ed..

[23] Abdelhamid M.A.S., Fábián L., MacDonald C.J., Cheesman M.R., Gates A.J., Waller Z.A.E. (2018). Redox-dependent control of i-Motif DNA structure using copper cations. Nucleic Acids Res..

[24] Park K.S., Jung C., Park H.G. (2010). “Illusionary” Polymerase Activity Triggered by Metal Ions: Use for Molecular Logic-Gate Operations. Angew. Chem. Int. Ed..

[25] Park K.S., Lee C.Y., Park H.G. (2016). Metal ion triggers for reversible switching of DNA polymerase. Chem. Commun..

[26] Petty J.T., Zheng J., Hud N.V., Dickson R.M. (2004). DNA-templated Ag nanocluster formation. J. Am. Chem. Soc..

[27] Cerretani C., Kanazawa H., Vosch T., Kondo J. (2019). Crystal structure of a NIR-Emitting DNA-Stabilized Ag-16 Nanocluster. Angew. Chem. Int. Ed..

[28] Liu S., Clever G.H., Takezawa Y., Kaneko M., Tanaka K., Guo X., Shionoya M. (2011). Direct Conductance Measurement of Individual Metallo-DNA Duplexes within Single-Molecule Break Junctions. Angew. Chem. Int. Ed..

[29] Wang Z.-G., Elbaz J., Willner I. (2011). DNA Machines: Bipedal Walker and Stepper. Nano Lett..

[30] Freeman R., Finder T., Willner I. (2009). Multiplexed Analysis of Hg^2+^ and Ag^+^ Ions by Nucleic Acid Functionalized CdSe/ZnS Quantum Dots and Their Use for Logic Gate Operations. Angew. Chem. Int. Ed..

[31] Tanaka T., Tengeiji A., Kato T., Toyama N., Shionoya M. (2003). A discrete self-assembled metal array in artificial DNA. Science.

[32] Kondo J., Tada Y., Dairaku T., Hattori Y., Saneyoshi H., Ono A., Tanaka Y. (2017). A metallo-DNA nanowire with uninterrupted one-dimensional silver array. Nat. Chem..

[33] Ono A., Kanazawa H., Ito H., Goto M., Nakamura K., Saneyoshi H., Kondo J. (2019). Novel DNA Helical Wire Containing Hg-II-Mediated T:T and T:G Pairs. Angew. Chem. Int. Ed..

[34] Lansdown A.B.G. (2002). Silver. I: Its antibacterial properties and mechanism of action. J. Wound Care.

[35] Ono A., Cao S., Togashi H., Tadhiro M., Fujimoto T., Machinami T., Oda S., Miyake Y., Okamoto I., Tanaka Y. (2008). Specific interactions between silver(I) ions and cytosine-cytosine pairs in DNA duplexes. Chem. Commun..

[36] Kondo J., Tada Y., Dairaku T., Saneyoshi H., Okamoto I., Tanaka Y., Ono A. (2015). High-Resolution Crystal Structure of a Silver(I)-RNA Hybrid Duplex Containing Watson-Crick-like C-Silver(I)-C Metallo-Base Pairs. Angew. Chem. Int. Ed..

[37] Urata H., Yamaguchi E., Nakamura Y., Wada S. (2011). Pyrimidine-pyrimidine base pairs stabilized by silver(I) ions. Chem. Commun..

[38] Funai T., Miyazaki Y., Aotani M., Yamaguchi E., Nakagawa O., Wada S., Torigoe H., Ono A., Urata H. (2012). AgI Ion Mediated Formation of a C-A Mispair by DNA Polymerases. Angew. Chem. Int. Ed..

[39] Funai T., Nakamura J., Miyazaki Y., Kiriu R., Nakagawa O., Wada S., Ono A., Urata H. (2014). Regulated Incorporation of Two Different Metal Ions into Programmed Sites in a Duplex by DNA Polymerase Catalyzed Primer Extension. Angew. Chem. Int. Ed..

[40] Goncharova I. (2014). Ag(I)-mediated homo and hetero pairs of guanosine and cytidine: Monitoring by circular dichroism spectroscopy. Spectrochim. Acta A.

[41] Swasey S.M., Leal L.E., Lopez-Acevedo O., Pavlovich J., Gwinn E.G. (2015). Silver(I) as DNA glue: Ag^+^-mediated guanine pairing revealed by removing Watson-Crick constraints. Sci. Rep..

[42] Swasey S.M., Gwinn E.G. (2016). Silver-mediated base pairings: Towards dynamic DNA nanostructures with enhanced chemical and thermal stability. New J. Phys..

[43] Liu H., Shen F., Haruehanroengra P., Yao Q., Cheng Y., Chen Y., Yang C., Zhang J., Wu B., Luo Q. (2017). A DNA Structure Containing Ag-I-Mediated G:G and C:C Base Pairs. Angew. Chem. Int. Ed..

[44] Swasey S.M., Rosu F., Copp S.M., Gabelica V., Gwinn E.G. (2018). Parallel Guanine Duplex and Cytosine Duplex DNA with Uninterrupted Spines of Ag-I-Mediated Base Pairs. J. Phys. Chem. Lett..

[45] Chen X., Makkonen E., Golze D., Lopez-Acevedo O. (2018). Silver-Stabilized Guanine Duplex: Structural and Optical Properties. J. Phys. Chem. Lett..

[46] Shukla S., Sastry M. (2009). Probing differential Ag^+^-nucleobase interactions with isothermal titration calorimetry (ITC): Towards patterned DNA metallization. Nanoscale.

[47] New S.Y., Lee S.T., Su X.D. (2016). DNA-templated silver nanoclusters: Structural correlation and fluorescence modulation. Nanoscale.

[48] Schultz D., Brinson R.G., Sari N., Fgan J.A., Bergonzo C., Lin N.J., Dunkers J.P. (2019). Structural insights into DNA-stabilized silver clusters. Soft. Matter..

[49] Phan A.T., Mergny J. (2002). -L. Human telomeric DNA: G-quadruplex, i-motif and Watson-Crick double helix. Nucleic Acids Res..

[50] Sirajuddin M., Ali S., Badshah A. (2013). Drug-DNA interactions and their study by UV-Visible, fluorescence spectroscopies and cyclic voltametry. J. Photochem. Photobiol. B.

[51] Miyake Y., Togashi H., Tashiro M., Yamaguchi H., Oda S., Kudo M., Tanaka Y., Konda Y., Sawa R., Fujimoto T. (2006). Mercury(II)-mediated formation of thymine-Hg-II-thymine base pairs in DNA duplexes. J. Am. Chem. Soc..

[52] Weiss J.N. (1997). The Hill equation revisited: Uses and misuses. FASEB J..

[53] Kypr J., Kejnovská I., Renčiuk D., Vorlíčková M. (2009). Circular dichroism and conformational polymorphism of DNA. Nucleic Acids Res..

[54] Tan W., Fang X., Li J., Liu X. (2000). Molecular beacons: A novel DNA probe for nucleic acid and protein studies. Chem. Eur. J..

[55] Østergaard M.E., Hrdlicka P.J. (2011). Pyrene-functionalized oligonucleotides and locked nucleic acids (LNAs): Tools for fundamental research, diagnostics, and nanotechnology. Chem. Soc. Rev..

[56] Okamoto A., Kanatani K., Saito I. (2004). Pyrene-Labeled Base-Discriminating Fluorescent DNA Probes for Homogeneous SNP Typing. J. Am. Chem. Soc..

[57] Yang C.J., Jockusch S., Vicens M., Turro N.J., Tan W. (2005). Light-switching excimer probes for rapid protein monitoring in complex biological fluids. Proc. Natl. Acad. Sci. USA.

[58] Nagatoishi S., Nojima T., Juskowiak B., Takenaka S. (2005). A Pyrene-Labeled G-Quadruplex Oligonucleotide as a Fluorescent Probe for Potassium Ion Detection in Biological Applications. Angew. Chem. Int. Ed..

[59] Hofstadler S.A., Griffey R.H. (2001). Analysis of Noncovalent Complexes of DNA and RNA by Mass Spectrometry. Chem. Rev..

[60] Bahr U., Karas M., Hillenkamp F. (1994). Analysis of biopolymers by matrix-assisted laser desorption/ionization (MALDI) mass spectrometry. Fresenius J. Anal. Chem..

[61] Gut I.G. (2004). DNA analysis by MALDI-TOF mass spectrometry. Hum. Mutat..

[62] Murphy M.C., Rasnik I., Cheng W., Lohman T.M., Ha T. (2004). Probing single-stranded DNA conformational flexibility using fluorescence spectroscopy. Biophys. J..

[63] Kang J., Jung J., Kim S.K. (2014). Flexibility of single-stranded DNA measured by single-molecule FRET. Biophys. J..

[64] Mergny J.-L., Lacroix L. (2003). Analysis of thermal melting curves. Oligonucleotides.

[65] Zhang D., Wang H. (2019). Fluorescence Anisotropy Reduction of An Allosteric G-Rich Oligonucleotide for Specific Silver Ion and Cysteine Detection Based on the G-Ag^+^-G Base Pair. Anal. Chem..

