# Supplementary material for: Intramolecular Folding of PolyT Oligonucleotides Induced by Cooperative Binding of Silver(I) Ions
Source: Molecules. 2022 Nov 14;27(22):7842. doi: 10.3390/molecules27227842 (PMC9694225; doi:10.3390/molecules27227842)
Supplement: Supplementary file 1 [file molecules-27-07842-s001.zip › molecules-1973426-supplementary.pdf]

Supporting Information

Intramolecular Folding of PolyT Oligonucleotides Induced by Cooperative Binding of Silver(I) Ions

Jinghua Hao[1,2,3], Dong Cao[2], Qiang Zhao[1,2,3], Dapeng Zhang[1,2,3,*] and Hailin Wang[1,2,3]

[1]School of Environment, Hangzhou Institute for Advanced Study, UCAS, Hangzhou, 310024, China

[2]State Key Laboratory of Environmental Chemistry and Ecotoxicology, Research Center for Eco-Environmental Sciences, Chinese Academy of Sciences, Beijing, 100085, China

[3]University of Chinese Academy of Sciences, Beijing, 100049, China

*Corresponding author: Dapeng Zhang

Fax: +86-10-62849600. Tel.: +86-10-62849611. E-mail: dpzhang@rcees.ac.cn.

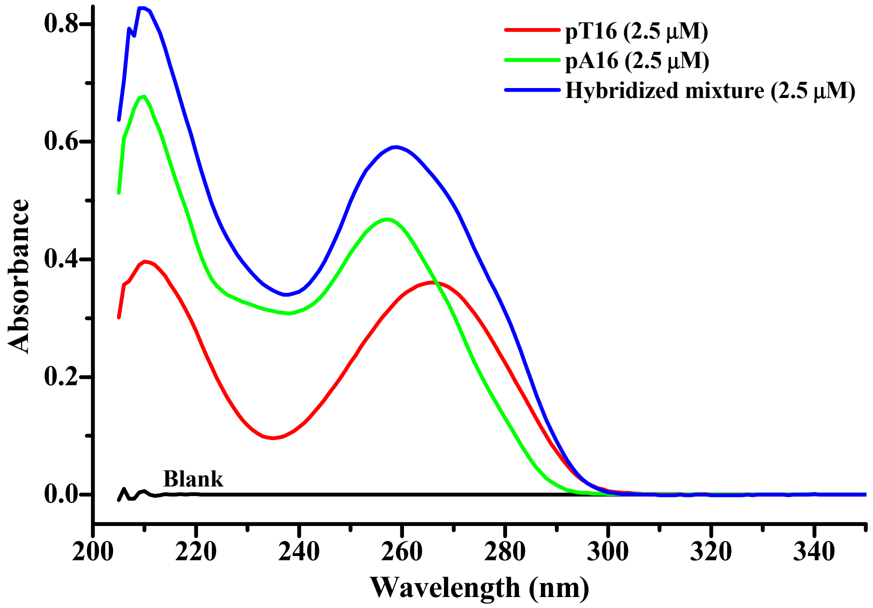

Figure S1 UV absorption spectra of 2.5 μM pT16 (red curve), 2.5 μM pA16 (green curve) and hybridized mixture (blue curve) of 2.5 μM pT16 with equimolar pA16. The samples were prepared in 10 mM Tris-HCl, 50 mM NaCl, 1 mM EDTA at pH 7.5.

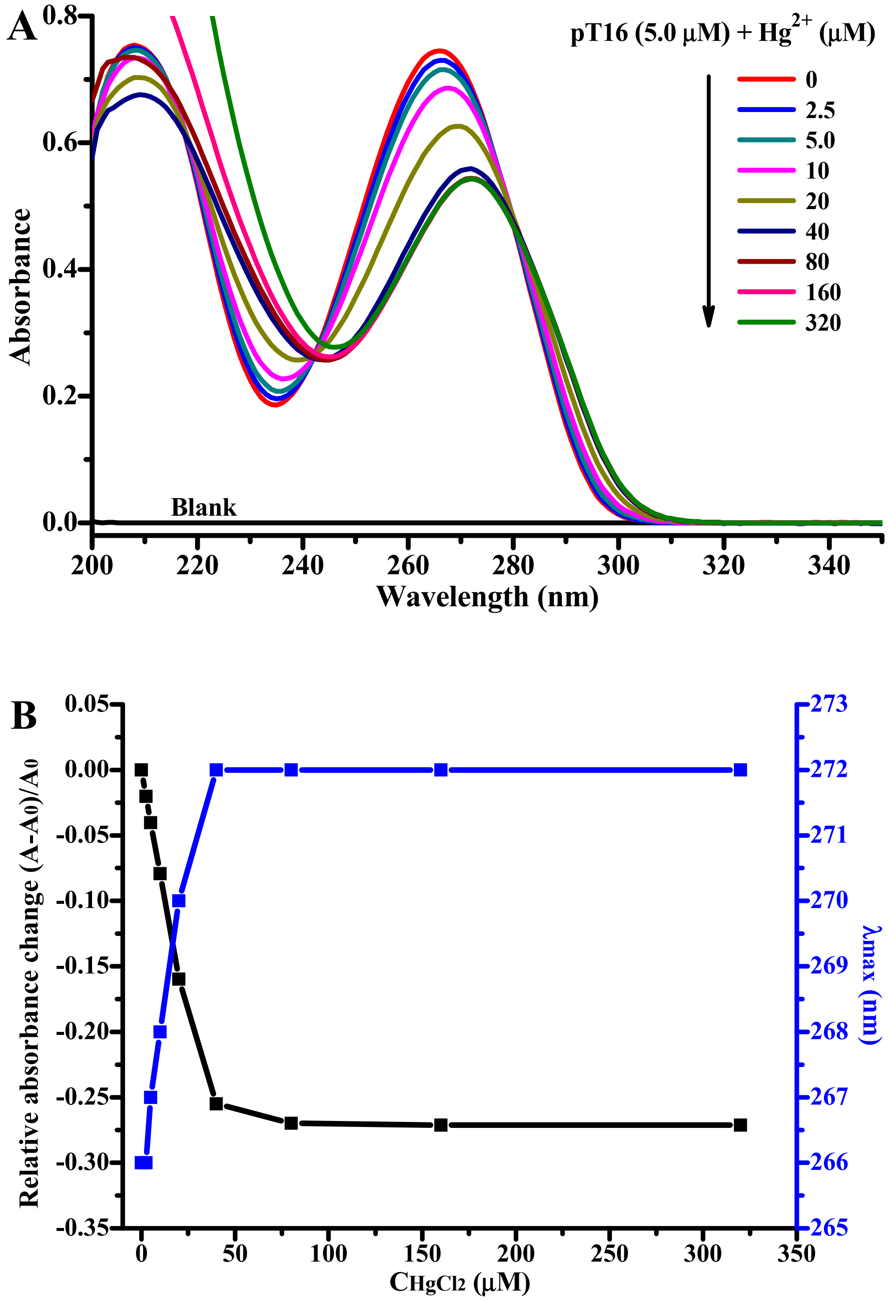

Figure. S2 (A) UV absorption spectra of 5.0 μM pT16 in the absence and presence of 2.5, 5.0, 10, 20, 40, 80, 160 and 320 μM $HgCl_2$. (B) Plots of the relative absorbance change (left, black) and wavelength (right, blue) at the maximum absorption against the concentration of $HgCl_2$ for pT16 solutions at 5.0 μM (square). The samples were prepared in 10 mM Tris-HAc buffer at pH 7.0.

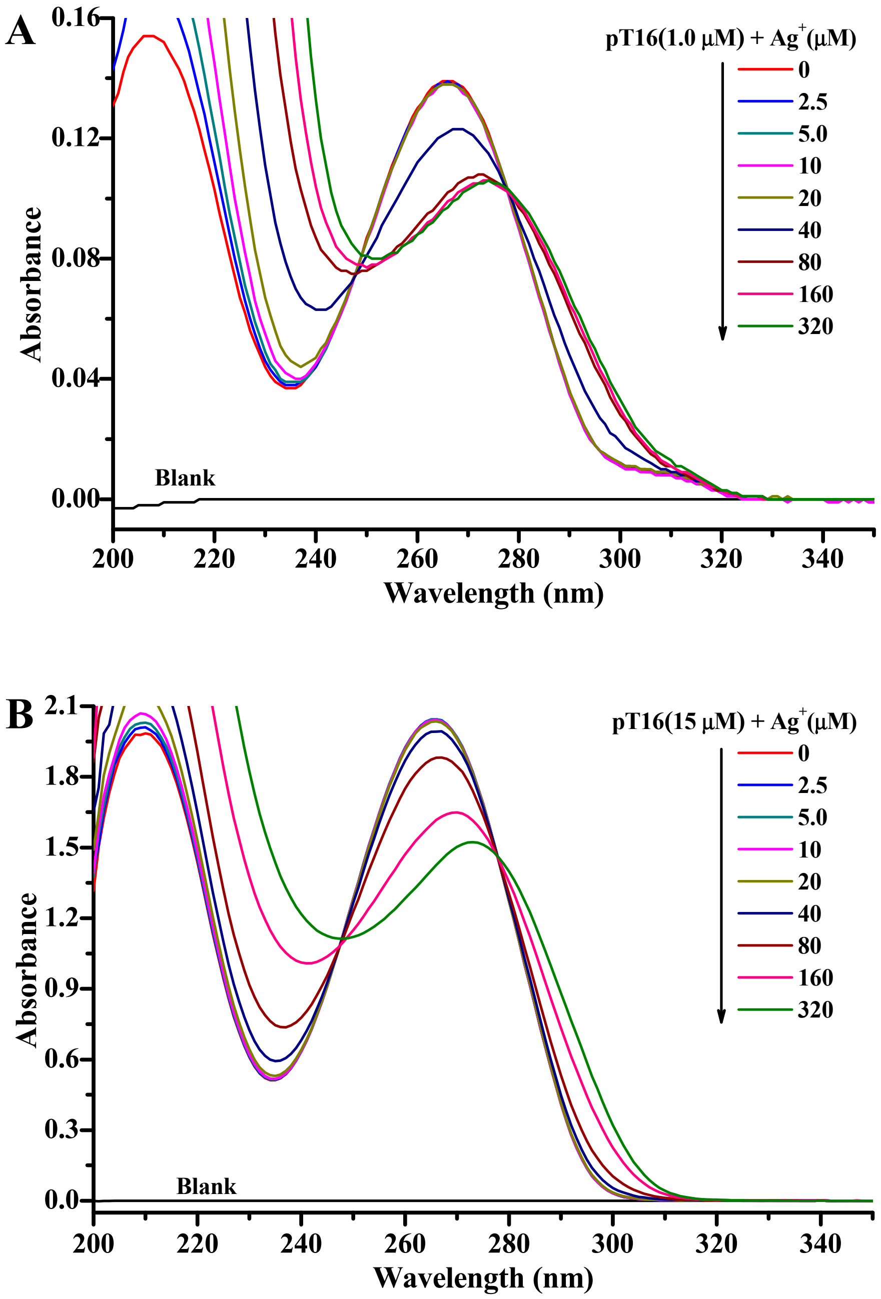

Figure.S3 UV absorption spectra of 1.0 μM (A) and 15 μM (B) pT16 in the absence and presence of 2.5, 5.0, 10, 20, 40, 80, 160 and 320 μM $AgNO_3$. The samples were prepared in 10 mM Tris-HAc buffer at pH 7.0.

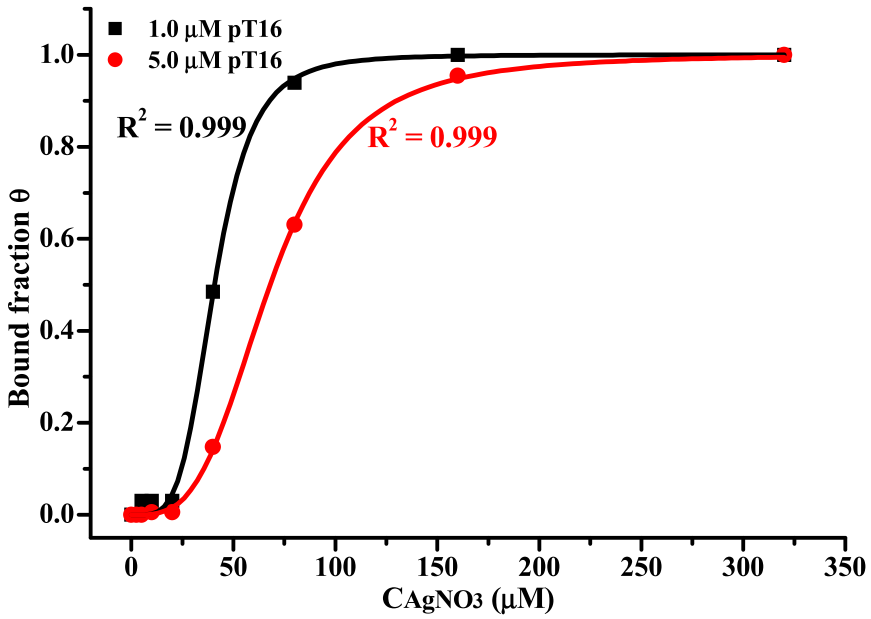

Figure. S4 Plots of the bound fraction against the concentration of $AgNO_3$ for 1.0 μM pT16 (black square) and 5.0 μM pT16 (red circle). The curves were obtained by fitting the experimental data with the Hill-Langmuir equation.

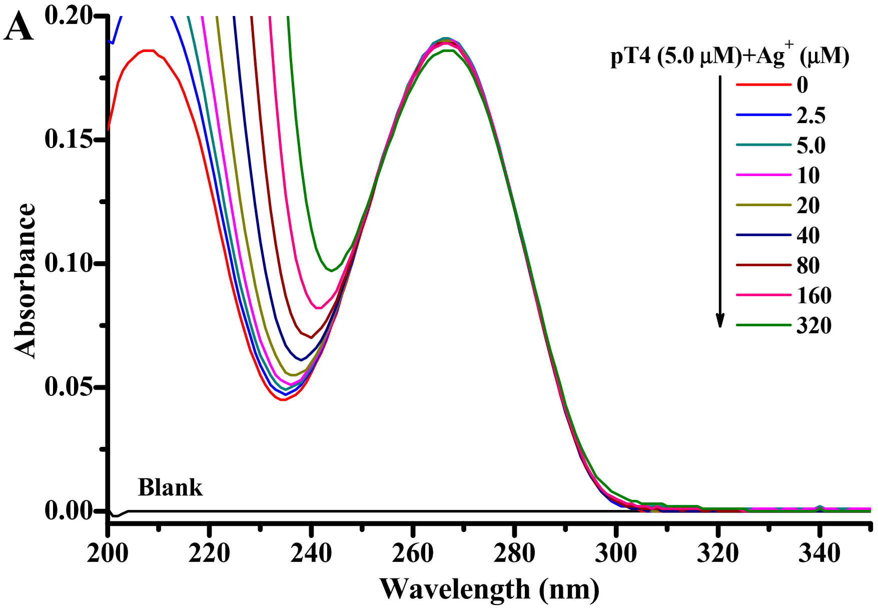
A
pT4 (5.0 μM)+Ag+ (μM)
0
2.5
5.0
10
20
40
80
160
320
Blank
Absorbance
Wavelength (nm)

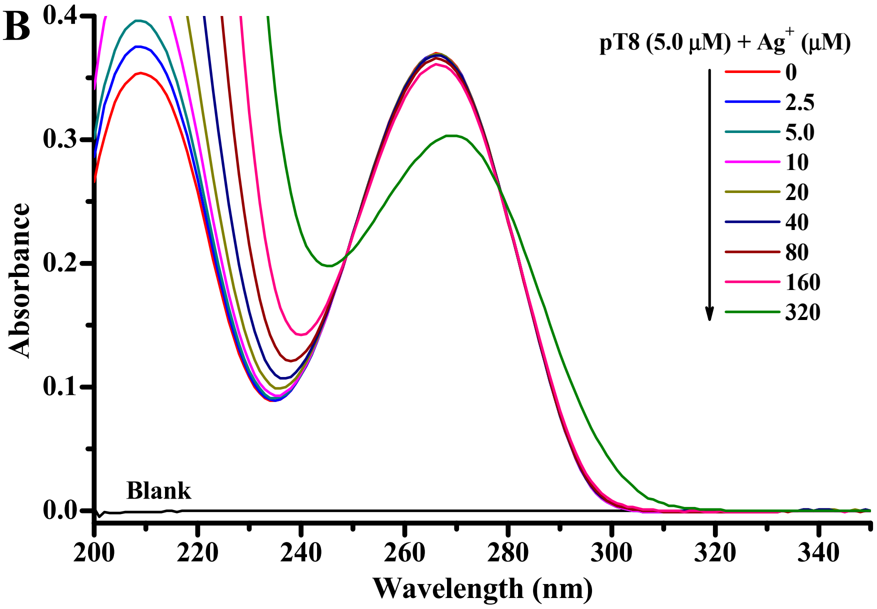
B
pT8 (5.0 μM) + Ag+ (μM)
0
2.5
5.0
10
20
40
80
160
320
Blank
Absorbance
Wavelength (nm)

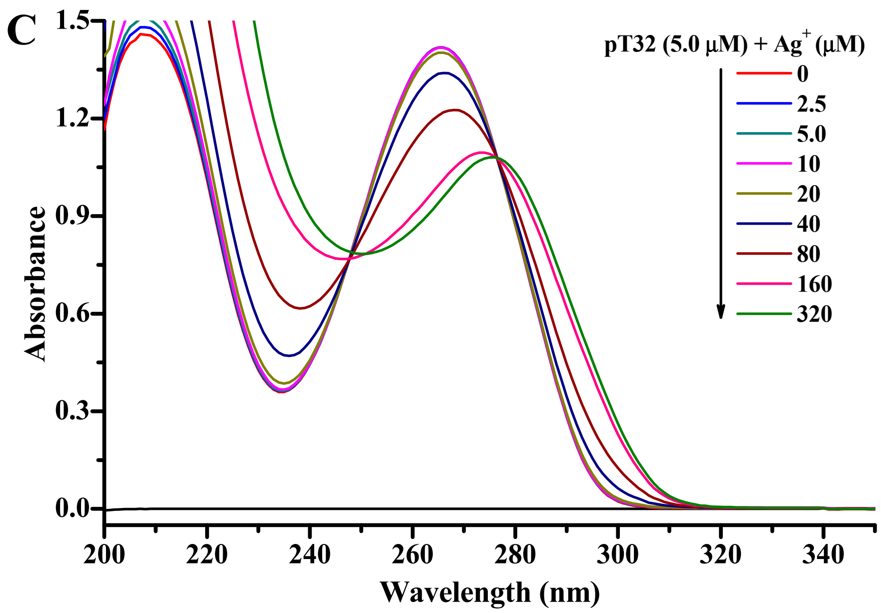

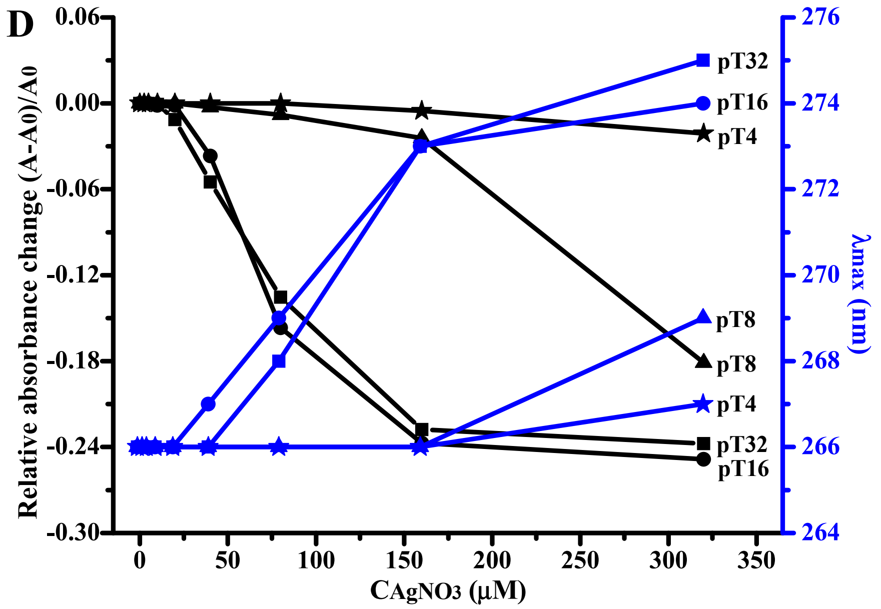

Figure. S5 UV absorption spectra of 5.0 μM pT4 (A), pT8 (B) and pT32 (C) in the absence and presence of 2.5, 5.0, 10, 20, 40, 80, 160 and 320 μM $AgNO_3$. (D) Plots of absorbance change (left, black) and wavelength (right, blue) at the maximum absorption against the concentration of $AgNO_3$ for 5.0 μM pT4 (star), pT8 (uptriangle), pT16 (circle) and pT32 (square). The samples were prepared in 10 mM Tris-HAc buffer at pH 7.0. A_0 and A are the absorbance at maximum absorption of oligonucleotide in the absence and presence of $AgNO_3$, respectively.

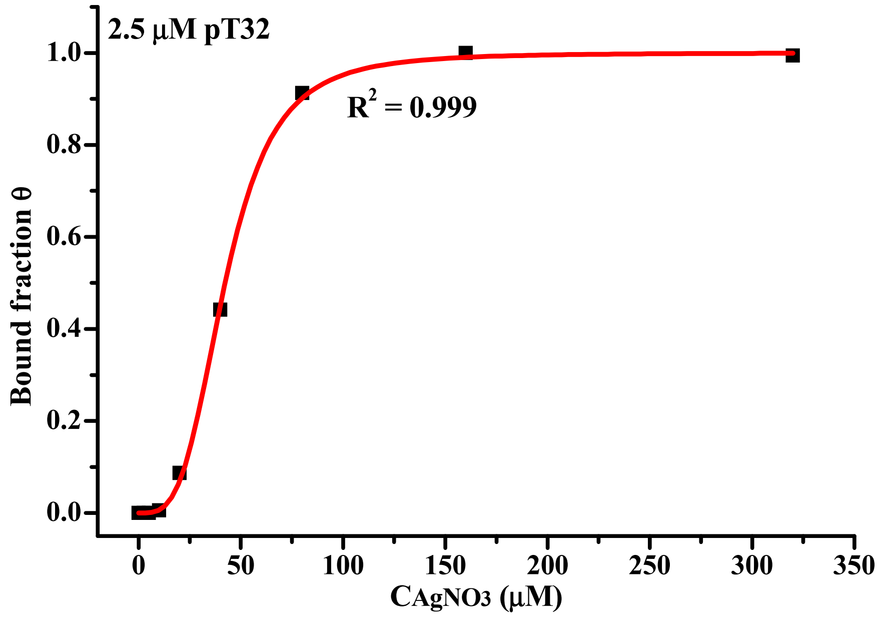

Figure. S6 Plots of the bound fraction against the concentration of $AgNO_3$ for 2.5 μM pT32. The curve was obtained by fitting the experimental data with the Hill-Langmuir equation.

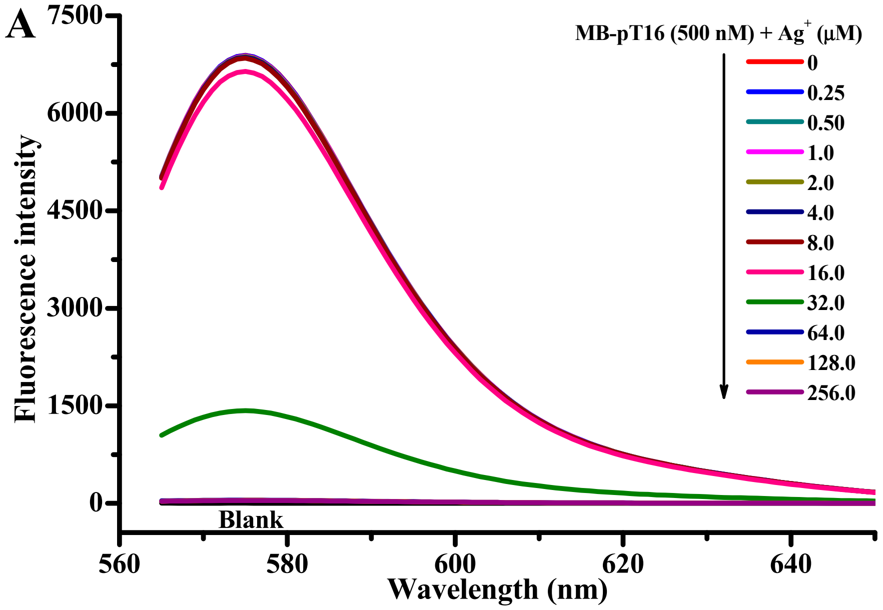

Figure. S7 Fluorescence emission spectra of 500 nM (A) and 2.5 μM (B) MB-pT16 in the absence and presence of 0.5-fold, 1-fold, 2-fold, 4-fold, 8-fold, 16-fold, 32-fold, 64-fold, 128-fold, 256-fold and 512-fold $AgNO_3$. The samples were prepared in 10 mM Tris-HAc buffer at pH 7.0. The excitation wavelength was set at 557nm. The excitation and emission slit bandwidths were set at 5 nm and 2.5 nm for 500 nM MB-pT16, and both set at 2.5 nm for 2.50 μM MB-pT16.

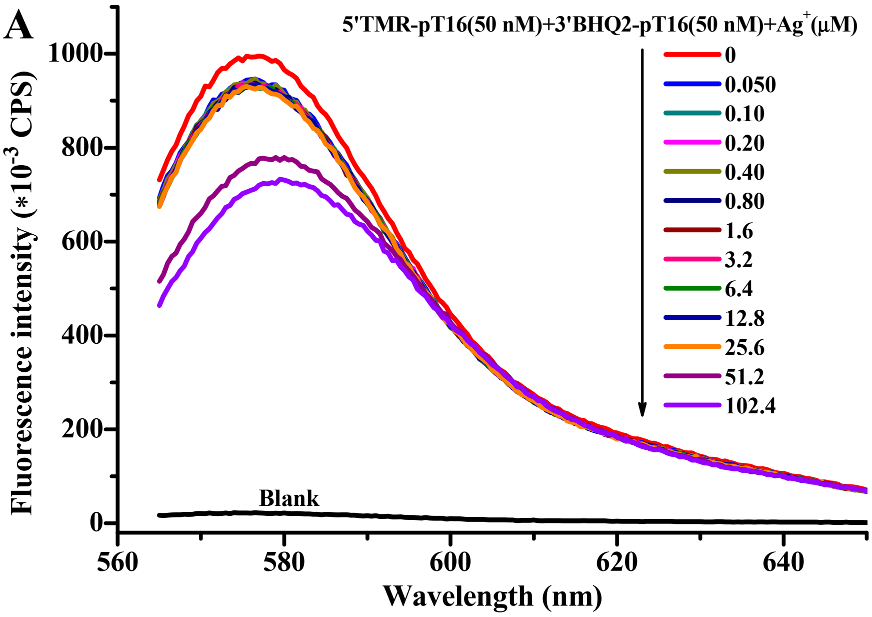
A
5'TMR-pT16(50 nM)+3'BHQ2-pT16(50 nM)+Ag+(μM)
0
0.050
0.10
0.20
0.40
0.80
1.6
3.2
6.4
12.8
25.6
51.2
102.4
Blank
Fluorescence intensity (*10-3 CPS)
Wavelength (nm)
0
200
400
600
800
1000
560
580
600
620
640

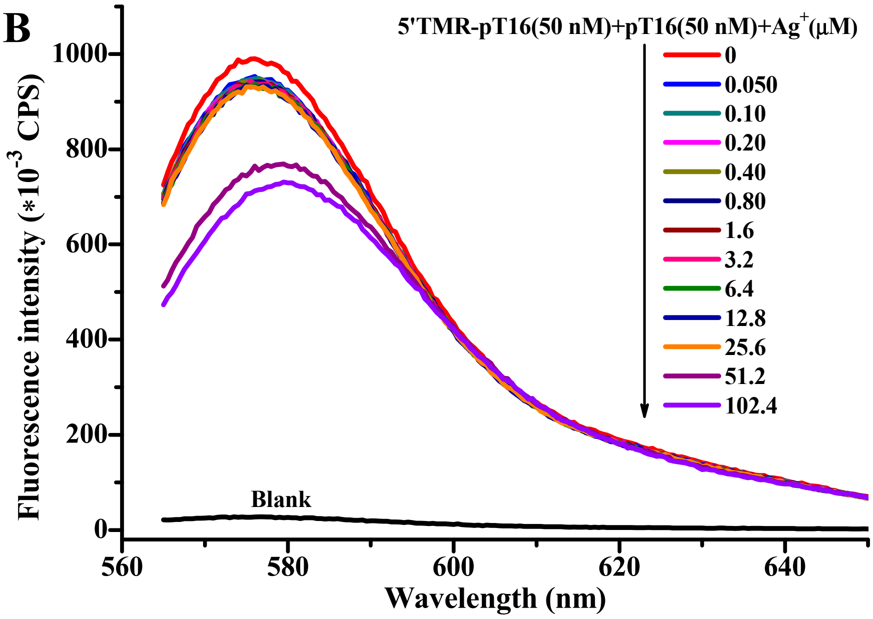
B
5'TMR-pT16(50 nM)+pT16(50 nM)+Ag+(μM)
0
0.050
0.10
0.20
0.40
0.80
1.6
3.2
6.4
12.8
25.6
51.2
102.4
Blank
Fluorescence intensity (*10-3 CPS)
Wavelength (nm)
0
200
400
600
800
1000
560
580
600
620
640

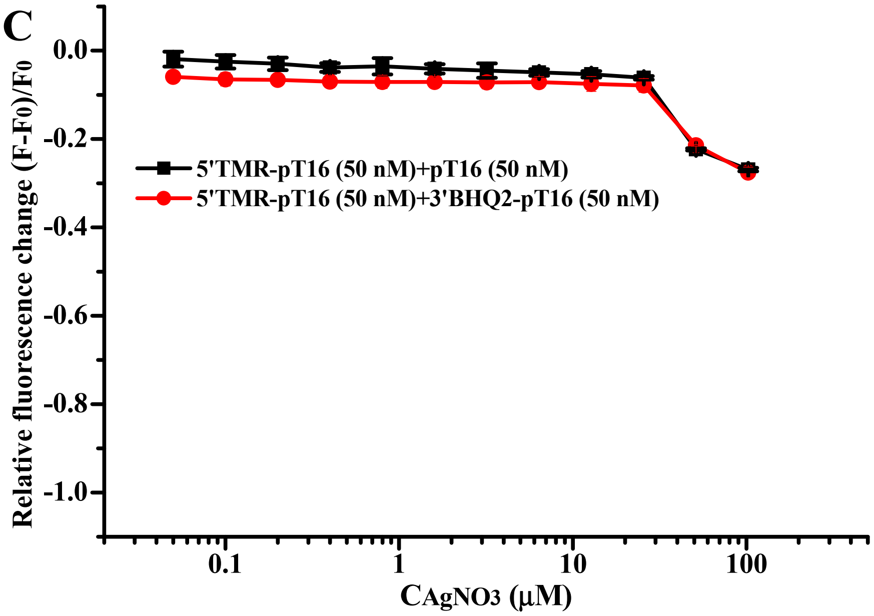

Figure. S8 Fluorescence emission spectra of the mixture of 50 nM 5’TMR-pT16 with equimolar 3’BHQ2-pT16 (A) or unlabeled pT16 (B) in the absence and presence of 0.050, 0.10, 0.20, 0.40, 0.80, 1.6, 3.2, 6.4, 12.8, 25.6, 51.2 and 102.4 μM $AgNO_3$. (C) Plots of the relative fluorescence change of TMR at 578 nm against the concentration of $AgNO_3$ for the mixture of 50 nM 5’TMR-pT16 with equimolar 3’BHQ2-pT16 (red circle) or unlabeled pT16 (black square). The samples were prepared in 10 mM Tris-HAc buffer at pH 7.0. The excitation wavelength was set at 557nm. The excitation and emission slit bandwidths were set at 5 nm and 2.5 nm. F_0 and F were the fluorescence intensity of 5’TMR-pT16 at 578 nm in the absence and presence of $AgNO_3$, respectively.

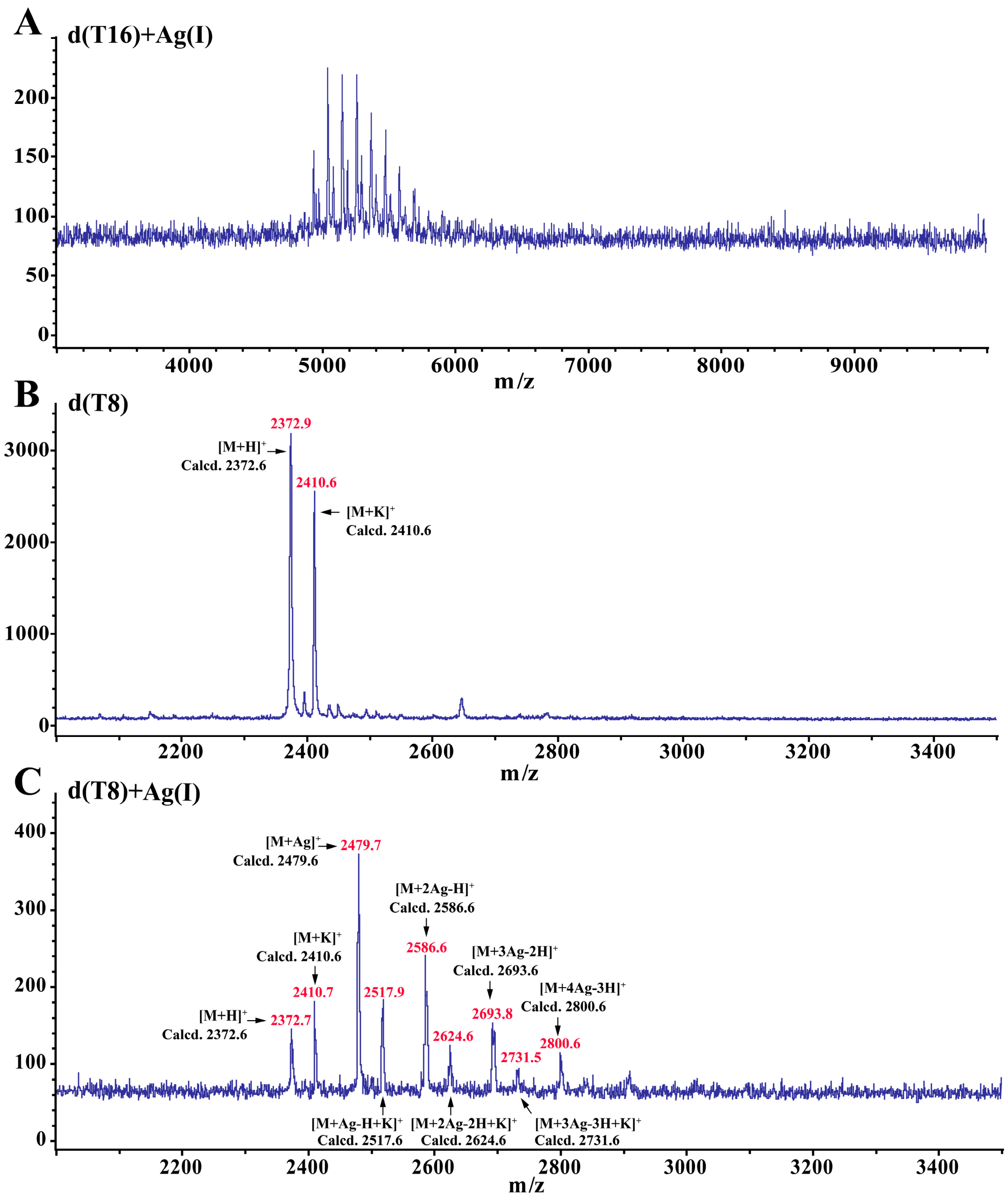

Figure. S9 Positive MALDI-TOF/MS spectra of 100 μM pT16 in the presence of 800 μM $AgNO_3$ in a wider molecular weight range (A) and 100 μM pT8 in the absence (B) and presence (C) of 400 μM $AgNO_3$. The samples were prepared in 10 mM Tris-HAc buffer at pH 7.0.

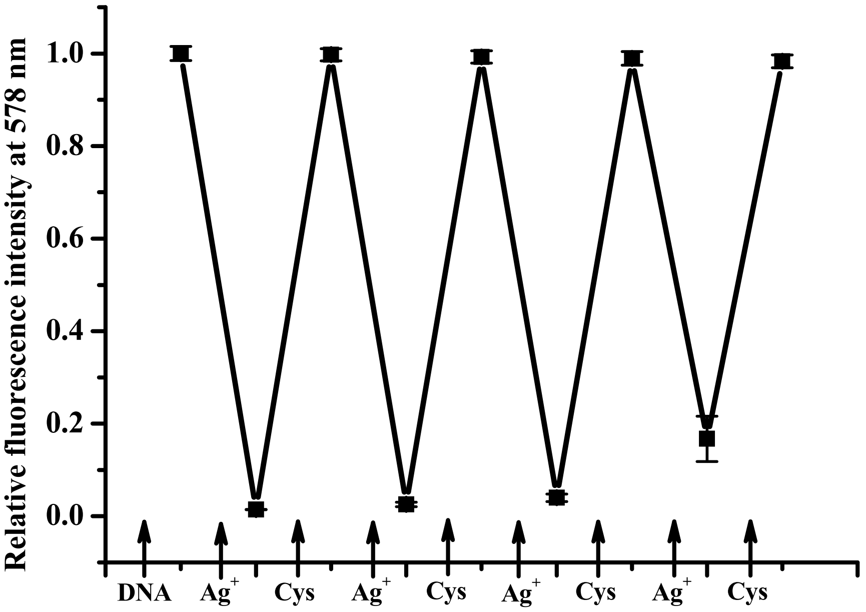

Figure. S10 Cycling of the folding and unfolding of 100 nM MB-pT16 through monitoring the relative fluorescence of TMR at 578 nm. The samples were prepared in 10 mM Tris-HAc buffer at pH 7.0. Arrows on the x-axis indicated the addition of 51.2 μM $AgNO_3$ or 25.6 μM L-Cysteine.
